# Acute Dilatation of the Heart

**Published:** 1898-07-09

**Authors:** 


### ACUTE DILATATION OF THE HEART.

The papers read by Dr. D. B. Lees and Dr. Poynton, at the Royal Medical and Chirurgical Society, on acute dilatation of the heart, draw attention to a condition of great importance, which, from the details given, seems to be of not infrequent occurrence. Dilatation is commonly associated with some obstruction to blood flow or some impediment to cardiac muscular action, but the statement now made is that an acute dilatation of the heart is a common occurrence in a rheumatic attack apart from obstruction, even when arthritis and pyrexia are slight in amount, and apart from distinct endocarditis and from pericarditis. Perhaps still more important is the corollary that such an acute dilatation, occurring in conjunction with pericarditis or endocarditis, might greatly intensify the gravity of the attack. The clinical evidence of the condition is derived from tracings of the cardiac dulness at different stages of a rheumatic attack. The first three sets of tracings shown were taken from young adults, suffering from subacute rheumatism with slight arthritis and little pyrexia with no evidence of pericarditis or distinct endocarditis. All the tracings showed the same phenomenon—a large extension of the dulness at first, with gradual reduction and final return to the normal. Others were shown illustrating the occurrence of acute dilatation in conjunction with pre-existing and with concurrent disease, and it was claimed that these tracings were sufficient to prove that acute dilatation of the heart was a feature of rheumatism which deserved more attention than it had hitherto received. It was suggested that the acute cardiac dilatation produced by rheumatism was analogous to the acute dilatation often resulting from influenza, and that in both cases it was due to the poisonous action of a microbic toxin on the cardiac muscle. The same thing was stated to occur in connection with the rheumatism and chorea of childhood. During the period of increase in the cardiac dulness the cardiac impulse became diffused and displaced to the left, the first sound also became weak and short, while the pulmonary second sound was accentuated. When, on the other hand, the dulness diminished, the impulse and the sounds became more normal. Clearly the difficulty will be to distinguish the condition described from effusion in the pericardium.

